# Endocrine profile dataset of fasting and normally eating young, healthy men and following activation of brain areas involved in ingestive behaviour

**DOI:** 10.1016/j.dib.2019.104676

**Published:** 2019-10-17

**Authors:** Janis M. Nolde, Sophia G. Connor, Arkan Al-Zubaidi, Jana Laupenmühlen, Marcus Heldmann, Kamila Jauch-Chara, Thomas F. Münte

**Affiliations:** a Department of Neurology, University of Lübeck, Ratzeburger Allee 160, 23562, Lübeck, Germany; b Department of Psychiatry, University of Lübeck, Ratzeburger Allee 160, 23562, Lübeck, Germany; c Department of Psychiatry and Psychotherapy, Christian-Albrechts-University, Niemannsweg 147, 24105, Kiel, Germany; d Institute of Psychology II, University of Lübeck, Ratzeburger Allee 160, 23562, Lübeck, Germany; e University of Western Australia, Perth, Australia; f Royal Perth Hospital, Perth, Australia

**Keywords:** Fasting, Endocrine, Adiponectin, Glucose metabolism, fMRI

## Abstract

Data includes endocrine data (adiponectin, ACTH, cortisol, C-peptide, insulin and glucose) of a 38 hour fasting intervention and a control condition with standardised meals in young healthy male subjects. The data was collected using a within-design approach. The data of ten common bilateral regions of interest (ROIs) involved in ingestive behaviour are included as fMRI percent signal change measurements of the amygdala, caudate nucleus, insula (classified into three regions), nucleus accumbens (NAcc), orbitofrontal cortex (OFC, classified into two different regions), pallidum, and lastly, the putamen. These measurements were performed whilst images of food were shown to participants during fMRI who would rate them on a scale from 1 to 8. Reaction times as well as each image's score are also included in the dataset. Endocrine data is especially useful as it is a well-controlled dataset of healthy young males in fasting and satiated conditions. Furthermore, this data can provide a physiological reference for experiments in patients with impaired glucose tolerance or metabolic syndrome. fMRI data may be useful as an extension of an existing dataset or for replication of the collected data.

**Table:** Specifications Table

Subject	Neuroscience: Endocrine and Autonomic Systems
Specific subject area	Crosstalk between the central nervous system and endocrine peripheral signals in ingestive behaviour.
Type of data	Tables
How data were acquired	Laboratory testing (Immunoassays and HemoCue® Glucose 201 DM Analyser) (Radiometer, Brønshøj, Denmark) fMRI (A 3 T Philips Achieva MR-scanner equipped with an 8 channel head-coil)
Data format	Raw
Parameters for data collection	Data was collected in a fasting and satiated condition. Participants were 18–30 years old, healthy males. There are 24 participants available for the endocrine data and 23 for fMRI related data as one participant was excluded due to movement artefacts.
Description of data collection	The collection of data started at 08:00 on the first day and blood samples were taken at 08:45, 10:00, 12:45, 14:00, 16:00, 18:00, 18:45, 20:00 and 22:00. On the second day, blood samples were taken at 08:45, 10:00, 11:45, 13:00. All participants underwent both conditions in randomised order exactly seven days apart. In the satiated conditions standardised meals were provided for all participants. The fMRI was performed at the end of the second day at 13:00.During the fMRI 72 pictures of food were presented each for 2 s and were rated on a scale of 1–8 by the participants.
Data source location	Institution: University of Lübeck, Institute for Neurology City/Town/Region: Lübeck Country: Germany
Data accessibility	With the article [1]
Related research article	Authors: Nolde, J. M., Laupenmühlen, J., Al-Zubaidi, A., Heldmann, M., Jauch-Chara, K., & Münte, T. F. Year: 2019 Title: Modulation of brain activity by hormonal factors in the context of ingestive behaviour Journal Metabolism: Clinical and Experimental DOI: https://doi.org/10.1016/j.metabol.2019.06.014

**Table:** 

**Value of the Data** • Healthy physiological responses to fasting interventions with thorough control condition • Data can be used as a control for experiments with fasting subjects • To develop further insight into the crosstalk between peripheral hormones and the central nervous system, potentially for other hormones or in an extended experimental setting • Thoroughly controlled physiological data, that can be used for new techniques of analysis or extension of large agglomerated datasets

## 1 Data

The following data files are included in the dataset.

The datafile “Perchange.dat” contains the activation of brain regions of interest (ROIs) as percent signal change values. Data table in long-format, as delimiters commas are used, as decimal separator dots are used. Data is differentiated by brain region, side, participant number, condition (fasting or control) and high or low rating defined by a median split of the rating data (in that order). The last column constitutes of the percent signal change data under the conditions outlined by the other fields of each row.

The “Hormones_fasting.txt” table contains the endocrine data (cortisol, ACTH, insulin, C-peptide, serum glucose) of the fasting condition. Data is presented in wide-format, semicolons are used as delimiter and dots as decimal separator. Columns are named after hormone, sample number (equivalent to time points of blood samples) and condition (B for fasting).

The “Hormones_normal_eating.txt” file contains endocrine data during the control condition with normal, standardised food consumption. Data table in wide-format, semicolons are used as delimiter and dots as decimal separator. Columns are named after hormone, sample number (equivalent to time points of blood samples) and condition (A for normal eating).

The “Rating_normal_eating.txt” file contains the rating of 72 images during the fMRI session in the control condition. Rating is expressed as numbers from 1 to 8 (high numbers indicate high likability of food shown in images). The data table is formatted in wide-format, ratings are present for all participants (rows) of 72 images in the fMRI session (A for normal eating).

The “Rating_fasting.txt” file contains the rating data for the fasting condition. Data table in wide-format, rating for all participants (rows) of 72 images in the fMRI session (B for fasting). The file “Reaction_time_rating.txt” contains a table in long-format of the reaction time until rating occurred, specified for subject, number of image and condition (A for normal eating and B for fasting).

## 2 Experimental design, materials, and methods

We enrolled 24 healthy, male participants without metabolic conditions and normal BMI (19–25 kg/m^2^). One subject had to be excluded due to motion artefact during the fMRI measurement. Medical histories and examinations were performed on all subjects before enrolment to rule out the presence of exclusion criteria such as consumption of more than 50 g of alcohol a week, use of any medication or cigarette smoking. Participants were also excluded if they were shift workers or high performance athletes.

The data was collected in two conditions in a within design. Every participant underwent both conditions in randomised order exactly seven days apart. In the fasting condition participants refrained from any energy intake (drinking only plain water) for 38 hours; in the other condition they received standardised meals at specified times (meals were prepared in exactly the same way with weighed ingredients). On day one meals were provided at 09:00 (breakfast: 2240 kcal, 14% protein, 46% fat and 40% carbohydrates), 13:00 (lunch: 1204 kcal, 17% protein, 31% fat and 52% carbohydrates) and 19:00 (dinner: 1199 kcal, 16% protein, 31% fat and 53% carbohydrates). On day two meals were provided at 09:00 (breakfast: 2240 kcal, 14% protein, 46% fat and 40% carbohydrates) and at 12:00 (lunch: 1174 kcal 18% protein, 31% fat and 50% carbohydrates).

Blood samples were taken on the first day at 08:45, 10:00, 12:45, 14:00, 16:00, 18:00, 18:45, 20:00 and 22:00; and on the second day at 08:45, 10:00, 11:45, 13:00. All samples were centrifuged (15 min with 2000×*g*) and stored at −80 °C.

Five hormones were measured using immunoassays (ACTH, C-peptide, insulin, cortisol, and adiponectin) at the same time to avoid inter-assay variability. The ACTH-assay (Roche Diagnostics, ELCIA, Indianapolis, IN, USA) had a measuring range of 0.220–440 pmol/L, an intra-assay coefficient of variation (CV) of <2.4% and an inter-assay CV of <4.2%. The C-peptide-assay had a measuring range of 0.003–13.3 nmol/L and an intra-assay CV of <5.0. The Insulin-assay had a measuring range of 1.39–6945 pmol/L, an intra-assay CV of <2.8% and an inter-assay CV of <4,9%. Cortisol had a measuring range of 0.5–1750 nmol/L, an intra-assay CV of <2.9% and an inter-assay CV of <4.7%. Adiponectin levels were measured with an Adiponectin ELISA (Immundiagnostik AG, Adiponectin total ELISA Kit, Bensheim, Germany) with an intra-assay CV of <3.4% and inter-assay CV of <6.3%. Glucose levels were determined with the HemoCue® Glucose 201 DM Analyser (Radiometer, Brønshøj, Denmark) immediately after the blood samples were taken.

Images in the fMRI setting were presented following a slow event-related design via monitor goggles in a randomised order. Images were presented every 20 seconds for a duration of 2 seconds. Participants rated the image after it had disappeared.

We used a 3 T Philips Achieva MR-scanner equipped with an 8 channel head-coil. A structural T1 weighted 3D turbo gradient Echo sequence with SENSE was performed with 180 sagittal slices of 1 mm, a 240 × 240 matrix and a flip angle of 9°. The echo time was 3.04 milliseconds (ms) with a repetition time of 6.72 ms. The functional session followed subsequently and consisted of 366 volumes. T2* weighted images were acquired with an Echo-planar pulse frequency with SENSE factor 2. Sagittal slices of 3 mm in a 64 × 64 matrix and a field of view of 192 mm and a flip angle of 80° were measured. The repetition time was 2 s and the echo time 25 ms. Percent signal change values were calculated for the most significant cluster with the function Rfxplot for SPM8 with Matlab 2015b and were used as the raw data [2].

## References

[1] Nolde J.M., Laupenmühlen J., Al-Zubaidi A., Heldmann M., Jauch-Chara K., Münte T.F. (2019). Modulation of brain activity by hormonal factors in the context of ingestive behaviour. Metabolism.

[2] Gläscher J. (2009). Visualization of group inference data in functional neuroimaging. Neuroinformatics.

